# Correction to “Alantolactone Induces Apoptosis in HepG2 Cells Through GSH Depletion, Inhibition of STAT3 Activation, and Mitochondrial Dysfunction”

**DOI:** 10.1155/bmri/9848297

**Published:** 2025-12-30

M. Khan, T. Li, M. Khan, et al., “Alantolactone Induces Apoptosis in HepG2 Cells Through GSH Depletion, Inhibition of STAT3 Activation, and Mitochondrial Dysfunction,” *BioMed Research International* 2013 (2013): 719858, https://doi.org/10.1155/2013/719858.

In the article, errors were identified in Figure 2. Specifically

• Panel a contains unexpectedly repeated elements to Panel e.
• Panel a contains unexpectedly repeated elements to Panel f.
• Panel e contains unexpectedly repeated elements to Panel f.

The correct Figure 2 is below:

Figure 2Changes in HepG2 cell morphology during alantolactone‐induced cell death. HepG2 cells were treated with 40 *μ*M alantolactone in the presence or absence of 3 mM NAC for various time intervals, and morphological changes were observed by phase contrast microscopy. (a) Control; (b–d) cells were treated with 40 *μ*M alantolactone for 3, 6, and 12 h; (e) cells were treated with 40 *μ*M alantolactone in the presence of 3 mM NAC for 12 h; and (f) cells were treated with NAC alone for 12 h, respectively. (g) Cells were treated with 40 *μ*M alantolactone as described above, and live and dead cells were quantified using fluorescent probe calcein AM and PI as described in Section 2. Data are expressed as mean ± SD (*n* = 3). Columns not sharing the same superscript letter differ significantly (*p* < 0.05).

**Figure:**
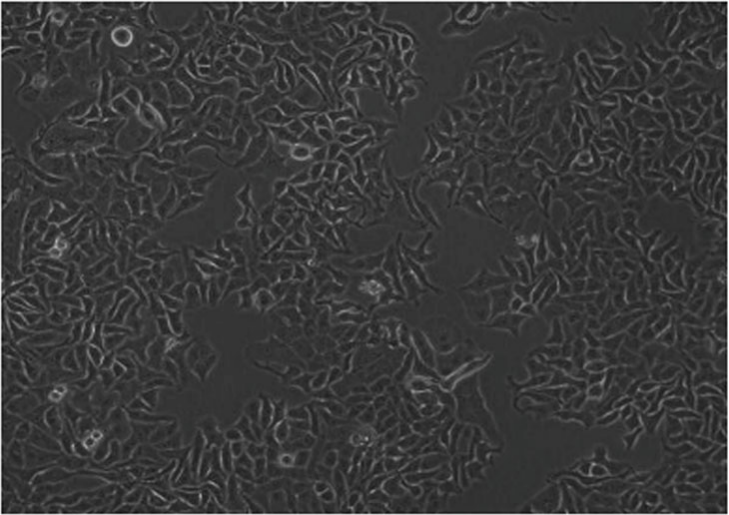
(a)

**Figure:**
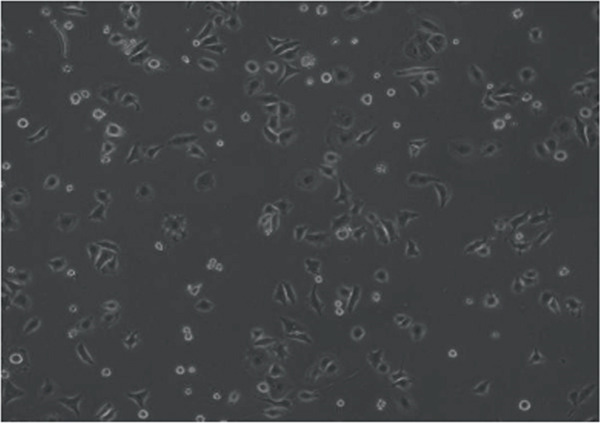
(b)

**Figure:**
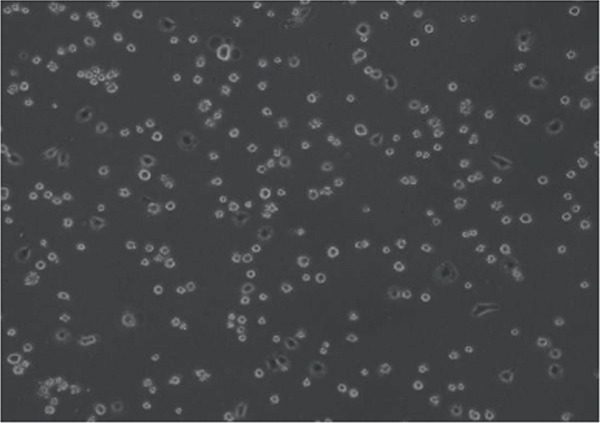
(c)

**Figure:**
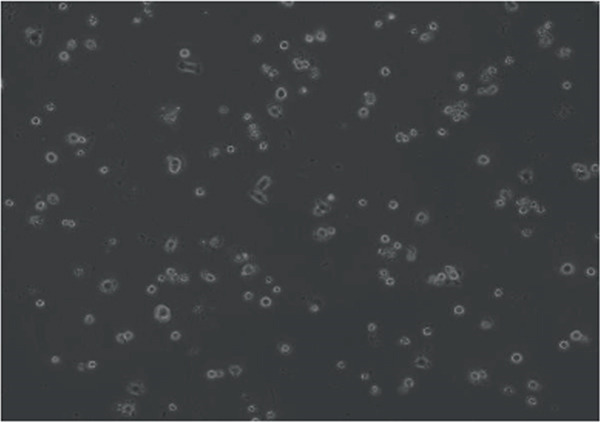
(d)

**Figure:**
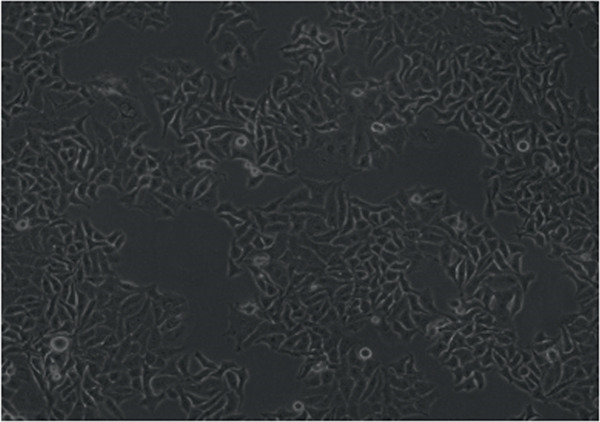
(e)

**Figure:**
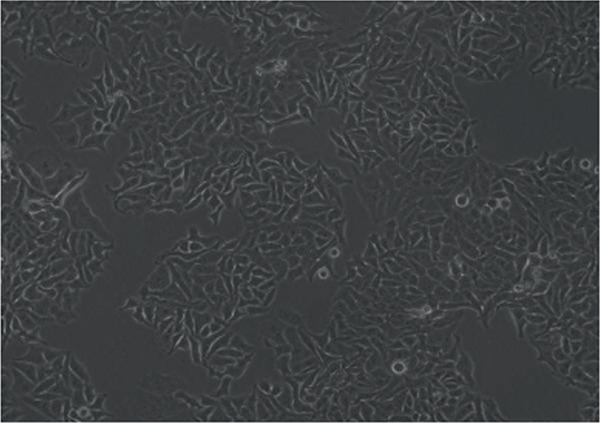
(f)

**Figure:**
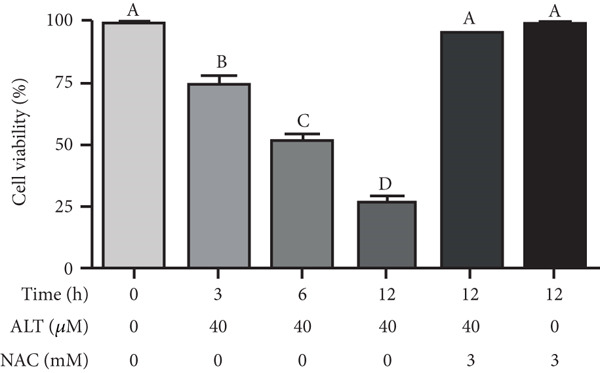
(g)

We apologize for these errors.

