# Supplementary material for: Alantolactone Induces Apoptosis in HepG2 Cells through GSH Depletion, Inhibition of STAT3 Activation, and Mitochondrial Dysfunction
Source: Biomed Res Int. 2012 Dec 27;2013:719858. doi: 10.1155/2013/719858 (PMC3591150; doi:10.1155/2013/719858)
Supplement: Supplementary file 1 — Purity of Alantolactone (99.12%) was determined by HPLC. HPLC were performed on a Waters XTerra C18 (4.6mm×150mm, 5?m). The mobile phase consisted of (A) acetonitrile containing 0.2% acetic acid and (B) 0.2% acetic acid in water with gradient elution: 0–15 min, 10–70% B; 15–30 min, 70–80% B; The temperature of autosampler was maintained at 25 ?C and detection wavelength was 254nm. [file 719858.f1.pdf]

Alantolactone Induces Apoptosis in HepG2 Cells Through GSH Depletion, Inhibition of STAT3 Activation and Mitochondrial Dysfunction.

Muhammad Khan[1,2], Ting Li[1], Muhammad Khalil Ahmad Khan[2], Azhar Rasul[1], Faisal Nawaz[3], Meiyan Sun[1], Yongchen Zheng[1*] and Tonghui Ma[1]

[1]Central Research Laboratory, Jilin University Bethune Second Hospital, Changchun 130041, P. R. China

[2] Department of Zoology, University of the Punjab, Quaid-e-Azam Campus Lahore 54590, Pakistan.

[3]Key Laboratory of Inorganic Synthesis and Preparative Chemistry, Jilin University Qianjin Street Changchun 130012, P. R. China

*Corresponding author:

Yongchen Zheng, Central Research Laboratory, Jilin University Bethune Second Hospital, Changchun, P. R. China. Tel/Fax: +86-431-88805200. E-mail: jlzyc@yahoo.com.cn

Abstract

Signal transducers and activators of transcription 3 (STAT3) constitutively expresses in human liver cancer cells and has been implicated in apoptosis resistance and tumorigenesis. Alantolactone, a sesquiterpene lactone, has been shown to possess anticancer activities in various cancer cell lines. In our previous report, we showed that alantolactone induced apoptosis in U87 glioblastoma cells via GSH depletion and ROS generation. However, the molecular mechanism of GSH depletion remained unexplored. The present study was conducted to envisage the molecular mechanism of alantolactone-induced apoptosis in HepG2 cells by focusing on the molecular mechanism of GSH depletion and its effect on STAT3 activation. We found that alantolactone induced apoptosis in HepG2 cells in a dose-dependent manner. This alantolactone-induced apoptosis was found to be associated with GSH depletion, inhibition of STAT3 activation, ROS generation, mitochondrial transmembrane potential dissipation, and increased Bax/Bcl-2 ratio and caspase-3 activation. This alantolactone-induced apoptosis and GSH depletion were effectively inhibited or abrogated by a thiol antioxidant, N-acetyl-L-cysteine (NAC). The data demonstrate clearly that intracellular GSH plays central role in alantolactone-induced apoptosis in HepG2 cells. Thus, alantolactone may become a lead chemotherapeutic candidate for the treatment of liver cancer.

Key words: Alantolactone, glutathione, STAT3, ROS, HepG2, apoptosis

Introduction

Hepatocellular carcinoma is currently the fifth most common cancer and third leading cause of cancer related-deaths in the world. Over 600000 patients die because of liver cancer in the world every year. Despite significant advances in surgery and chemotherapy, the majority of patients with hepatocellular carcinoma die within one year of diagnosis [1-4]. At present, the hepatocellular carcinoma is mainly treated with surgery and chemotherapy [5, 6]. Currently, doxorubicin is the most widely used drug against liver cancer either as single agent or in combination with other chemotherapeutics like cisplatin. However, the outcomes of the existing conventional chemotherapeutic drugs remain considerably low due to their severe toxicity on normal hepatocytes [7, 8]. Therefore, searching for highly efficient anticancer drugs with low hepatotoxicity remains a hot research area.

A causal link between chronic inflammation and development of cancer is well established. Many transcription factors such as NF-κB and STAT3 are key to innate inflammation. The constitutive activation of Signal Transducers and Activators of Transcription 3 (STAT3) has been frequently detected in many types of human cancers such as glioblastoma, myeloma, colorectal, and hepatocellular carcinoma where it plays important role in cell proliferation, tumor invasion, metastasis, and drug resistance [9, 10] . Therefore, blockage of STAT3 may have therapeutic potential in preventing and treating these cancers.

Sesquiterpene lactones are plant-derived bioactive constituents often used in traditional medicines against inflammation and cancer [11, 12]. Alantolactone, a sesquiterpene lactone component of *Anula helenium* and *Inula racemosa* has been shown to exhibit multiple pharmacological activities including anticancer effect [13, 14]. In our previous report, we have shown that alantolactone induces apoptosis in U87 glioblastoma cells via GSH depletion and mitochondrial dysfunction. However, the molecular mechanism of GSH depletion by alantolactone remained largely unknown. Furthermore, we showed that alantolactone did not induce hepatotoxicity and nephrotoxicity in mice[15]. Additionally, Butturini et al., showed that GSH depletion is involved in inhibition of STAT3 activation [16].

Keeping in mind the anti-inflammatory effect and GSH depleting activity of sesquiterpene lactones, we hypothesized that alantolactone can inhibit STAT3 activation and induce apoptosis in HepG2 cells. To evaluate this, we investigated the effect of alantolactone on GSH depletion and STAT3 and its downstream target gene Bcl-2 expressions. The data demonstrated that alantolactone induced apoptosis in HepG2 cells via GSH depletion, STAT3 inhibition, modulation of Bcl-2 family proteins and caspase-3 activation.

Materials and Methods

Alantolactone was obtained from Tauto Biotech Co., Ltd. (Shanghai, China) and purity (> 99%) was determined by HPLC (Supplementary data, Figure 1). Propidium iodide (PI) calcein acetoxymethylester (Calcein AM), Rhodamine 123, Dimethyl Sulfoxide (DMSO), MTT, Dulbecco's Modified Eagle's Medium (DMEM), fatal bovine serum (FBS), penicillin and streptomycin were purchased from Sigma (Beijing, China). Apoptosis assay kit was purchased from KeyGen (Shanghai, China) while Reactive oxygen species kit, and GSH/GSSG assay kit were purchased from Beyotime Institute of Biotechnology (Haimen, Jiangsu, China). Antibodies specific to Bax, Bcl-2, Caspase-3 and β-actin were obtained from Beyotime while ptyr705-STAT3 was obtained from Wuhan Boster Biological Technolgy, Ltd. (Wuhan, China). Antibody specific to glutathione reductase was obtained from BIOSS Beijing Biosynthesis Biotechnology, Co., LTD. Horseradish peroxidase-conjugated secondary antibodies (goat-anti-rabbit, goat-anti-mouse) were purchased from Sigma Aldrich.

Cell culture and treatment

The human HepG2 cells were obtained from Shanghai Cell Bank, China and were cultured in Dulbecco's Modified Eagle's Medium (DMEM) supplemented with 10% fatal

bovine serum (FBS), 100 units/mL penicillin and 100 μg/mL streptomycin and maintained at 37°C with 5% CO_2 in humidified atmosphere. Cells were treated with alantolactone dissolved in DMSO with a final DMSO concentration of 1%. DMSO treated cells were used as control in all the experiments.

Determination of cell viability

Cell viability was determined by MTT assay as described by us previously [17]. Briefly HepG2 cells were treated with different concentrations of alantolactone for 12 h. Following treatment, the MTT reagent was added (500μg/mL) and cells were further incubated at 37°C for 4 h. Subsequently 150 μL DMSO was added to dissolve farmazan crystals and absorbance was measured at 570 nm in a microplate reader (Thermo Scientific). The percentage of cell viability was calculated as follows:

Cell viability (%) = $(A570_{sample} - A570_{blank}) / (A570_{control} - A570_{blank}) \times 100$

The IC_{50} Values were calculated using GraphPad Prism 5.

Observation of morphological changes

HepG2 cells were treated with 40 μM alantolactone in the presence or absence of NAC for 0, 3, 6, and 12 h. Cell morphological changes were observed by phase contrast microscopy (Olympus 1x71).

Live/Dead assay

HepG2 cells were treated with 40 μM alantolactone in the presence or absence of NAC for 0, 3, 6, and 12 h. Live and dead cells were quantified using fluorescent probe calcein AM and PI and fluorescence microscope as described by us previously [18]. Celcein AM is cell membrane permeable and stains only viable cells whereas PI is cell membrane impermeable and stains only dead cells. Treated and untreated cells were collected, washed with phosphate buffered saline (PBS) and incubated with PBS solution

containing 2 µM calcein AM and 4 µM PI in the dark for 20 min at room temperature. Finally 100 cells were counted microscopically for the percentage of live and dead cells.

Apoptosis assay by Annexin V-FITC and propidium iodide (PI) staining

HepG2 cells were treated with 40 µM alantolactone for 0 , 3, 6, and 12 h. After treatment, cells were harvested, washed with PBS, and resuspended in 500 µL of binding buffer containing 5 µL Annexin V and 5 µL PI and put in the dark for 15 min according to the kit instructions (KeyGen, Shanghai, China). After incubation, samples were immediately analyzed by flow cytometry (Beckman Coulter, Epics XL).

Measurement of Reactive Oxygen Species (ROS)

The intracellular changes in ROS generation were measured by staining the cells with 2', 7' -dichlorofluorescein-diacetate (DCFH-DA) as described previously [18]. The fluorescent dye DCFH-DA is a cell membrane permeable and is converted into cell membrane impermeable nonfluorescent compound DCFH by intracellular esterases. Oxidation of DCFH by reactive oxygen species produces a highly fluorescent DCF. The fluorescence intensity of DCF inside the cells is proportional to the amount of peroxide produced. Briefly hepG2 cells were treated with 40 µM alantolactone for 0, 3, 6, and 12 h. After treatment, cells were further incubated with 10 µmol/L DCFH-DA at 37°C for 30 min. Subsequently cells were harvested, rinsed, re-suspended in PBS, filtered with 300 apertures and analyzed for 2', 7' -dichlorofluorescein (DCF) fluorescence by flow cytometry.

Measurement of Mitochondrial Membrane Potential (MMP)

Rhodamine 123 was used to evaluate the changes in mitochondrial membrane potential as described previously [18]. Briefly HepG2 cells were incubated with 40 µM

Alantolactone for 0, 3, 6, and 12 h. Following incubation, cells were collected, resuspended in 1mL PBS solution containing 10 µg Rhodamine 123 and incubated in the dark for 30 min. After incubation, cells were centrifuged at 500×g for 5 min, supernatant was removed, and pellet was gently rinsed with PBS once and then resuspended in 500µL PBS. After filtration (300 apertures), the suspension was analyzed by flow cytometry.

Measurement of GSH and GSSG

The intracellular reduced (GSH) and oxidized (GSSG) glutathione was determined spectrophotometrically using GSH and GSSG assay kit (Beyotime). Briefly, U87 cells were treated with 40 µM alantolactone for 0, 3, 6, and 12 h or with 40 µM alantolactone in the presence or absence of 2 mM methionine and 3mM NAC for 6 h. Following treatment, the intracellular and extracellular (medium) GSH and GSSG were measured according to the instruction of kit. The values were expressed as nmols GSH/mg protein.

HPLC Analysis

One mM alantolactone was incubated with 0, 5, 15 and 30 mM GSH in DMEM medium without FBS at 37°C for 30 min. Following incubation, the samples were analyzed by HPLC (Waters) using XTerra MS C18 (5µm, 4.6×150 mm) column. The mobile phase was composed of acetonitrile (A) and water (B). The gradient program was as follows: 0-30 min, A = 65%, B = 35%. The elution profile was analyzed at 227 nm by UV detector.

RNA Isolation and Semi quantitative RT-PCR

Total RNA was isolated from treated and untreated hepG2 cells using AxyPrep Multisource Total RNA Miniprep kit. cDNA was reverse-transcribed from 500 ng of total RNA in a final volume of 10 μl using PrimeScript RT reagent Kit (TakaRa, DRR037A), in accordance with the manufacturer’s instructions. This was followed by 35 cycles of (94 8C: 1 min; 52 8C: 30 sec; 72 8C: 1 min) and a final extension of 72 8C for 10 min. PCR product was visualized on 1% agarose gel containing ethidium bromide. The primers used were as follows:

γ-GCS, 5′-GGCACAGGTAAAACCAAATAGTAAC-3′ (Forward) and 5′-CAAATTG-TTTAGCAAATGCAGTCA-3′ (Reverse); GAPDH, 5′-ATGACATCAAGAAGGTGG-TG-3′ (Forward) and 5′- CATACCAGGAAATGAGCTTG-3′ (Reverse).

Immunobloting

Proteins were isolated from control and alantolactone-treated cells as described previously [18]. 40 μg proteins were electrophoresed on 12% SDS-PAGE and transferred to PVDF membrane. After blocking with 5% (w/v) non-fat milk and washing with Tris-buffered saline-Tween solution (TBST), membranes were incubated for 2 h at room temperature with Bax (1:300), BCL-2 (1:1000), Caspase-3 (1:500), $pTyr^{705}$ STAT3 (1:300), Glutathione Reductase (GR) (1:300), and β-actin (1:400) antibodies respectively. After washing, the blots were incubated with horse radish peroxidase conjugated goat anti-rabbit IgG or goat anti-mouse IgG secondary antibodies (1:5000) for 1 h at room temperature. After washing with TBST, signals were detected using ECL plus chemiluminescence kit (Millipore Corporation) on X-ray film.

Statistical Analysis

The results are expressed as Mean ± SD and statistically compared with control group or within the groups using one way ANOVA followed by Tukey's Multiple Comparison Test.

Results

Alantolactone inhibits growth of HepG2 cells *in vitro*

The effect of alantolactone on the growth of HepG2 cells was evaluated by MTT assay. Treatment with alantolactone for 12 h inhibited the growth of cells in a dose-dependent manner (Fig. 1). The IC_{50} value of alantolactone was 33 μM after 12 h treatment. 40 μM concentration was selected for the following experiments.

Microscopic study of HepG2 cells

To examine the effect of alantolactone on cell morphology, HepG2 cells were treated with 40 µM alantolactone for various time intervals (3, 6, & 12 h) and morphological changes were observed by phase contrast microscopy. The data showed that alantolactone induced severe morphological changes of cell death including rounding and shrinkage of cells, in a time-dependent manner (Fig. 2). Pretreatment of cells with 3 mM NAC, a specific ROS inhibitor, completely protected the cells from cytotoxic effect of alantolactone. Furthermore, live and dead cells were quantified using fluorescent probes calcein AM and PI. As shown in Fig. 2G, alantolactone treatment reduced the viability of cells in a time-dependent manner. The viability of cells treated with 40 µM alantolactone for 3, 6, and 12 h was 74.33%, 51.6%, and 27% respectively. These values were significantly lower than those of the control group (98.5%, $P<0.05$). Pretreatment of cells with 3 mM NAC reversed the cytotoxic effect of alantolactone indicating that alantolactone exerts cytotoxic effect through generation of ROS. However, NAC alone at this concentration did not affect the viability of cells as shown in Fig 2G.

Alantolactone induces apoptosis in hepG2 cells

The effect of alantolactone on cell apoptosis was evaluated by using annexin V-FITC/PI staining and flow cytometry. Translocation of phosphatidylserine (PS) to the outer leaflet of cellular membrane is the key step in the early stages of apoptosis. Annexin V selectively binds to PS and helps to identify cells undergoing apoptosis. When cells are double stained with Annexin V/PI, three different populations of cells can be observed. The cells that do not stain with either annexin V or PI are alive and reside in region B3; the cells that stain with only annexin V are in the stage of early apoptosis and

reside in region B4 while the cells that stain with both reagents are nonviable late apoptotic/necrotic cells and scatter in region B2.

Flow cytometric analysis of apoptosis showed that alantolactone induced apoptosis in HepG2 cells in a time-dependent manner as shown in Figure 3. Moreover, the early and late apoptosis process was also time-dependent. Treatment with 40 μM alantolactone for 3 and 6 h only increased the early apoptosis while at 12 h late apoptosis was also observed. The data suggested that alantolactone-induced cell death was through early apoptosis within 6 h, however, with the increase of time the late apoptotic rate also increased. Next, we treated the cells with 40 μM alantolactone for 12 h in the presence of 3 mM NAC and apoptosis rate was determined by flow cytometry. The data showed that NAC reversed the apoptotic effect of alantolactone indicating that alantolactone exerts apoptotic effect in HepG2 cells through generation of ROS (Fig 3E).

Alantolactone induces increased generation of ROS in HepG2 cells

Intracellular ROS generation in hepG2 cells was measured by flow cytometry using DCFH-DA. The data demonstrated that the level of ROS in cells treated with 40 μM alantolactone for 3, 6, and 12 h was 25%, 42% and 54% respectively (Fig. 4). These values were significantly higher than those of control group (15%, $P<0.05$)

Alantolactone disrupts mitochondrial membrane potential (MMP) in HepG2 cells

Depolarization in MMP is a characteristic feature of apoptosis. Excessive intracellular ROS production has been shown to induce apoptosis by disrupting MMP [19, 20] To investigate the role of ROS in alantolactone-induced apoptosis, we determined MMP in HepG2 cells using Rhodamine 123 and flow cytometry. The data showed that

alantolactone reduced the MMP in HepG2 cells in a time-dependent manner. As shown in Figure 5, MMP in cells treated with 40 μM alantolactone for 3, 6, and 12 h was significantly lower (85%, 79%, & 62% *vs* 98% in control group, P<0.05).

Alantolactone reduces intracellular GSH in HepG2 cells

Intracellular GSH plays major roles in the maintenance of redox status and defense of oxidative stress. GSH depletion is an early hallmark observed in ROS mediated apoptosis. We therefore investigated the status of intracellular GSH in control and alantolactone-treated cells. Time-dependent study revealed that GSH depletion was significant from 3 h of treatment and increased over time (Fig 6A). Over production of ROS can oxidize GSH into GSSG. We therefore, measured the level of GSSG in treated and untreated cells. As shown in Figure 6B, no change in the level of GSSG in control and alantolactone-treated cells was found.

Alantolactone depletes intracellular GSH via direct conjugation with GSH

The intracellular GSH depletion might be resulted either from increased intracellular oxidation of GSH or stimulated GSH extrusion through specific carrier or inhibition of GSH synthesis. In addition, sesquiterpene lactones contain α-methylene-β-lactone moiety which is highly reactive with cellular thiols and can deplete GSH by conjugating with sulfhydral group [21]. To shed light on the mechanism accounting alantolactone-mediated GSH depletion in HepG2 cells, we measured the concentration of GSH and GSSG in the culture medium. The concentration of GSH and GSSG in culture medium was not detectable. Over production of ROS can oxidize GSH into GSSG. Therefore, we incubated the cells with PEG-catalase and PEG-SOD and measured the level of GSH in cells. Pretreatment of cells with PEG-catalase and PEG-SOD alone or in combination did not prevent GSH depletion indicating that GSH depletion by alantolactone is not due to oxidation of GSH into GSSG (Figure 7A). During oxidative stress, GSH is oxidized to GSSG which is catalytically reduced back to GSH by

glutathione reductase (GR). Next we measured the expression of GR by Western blot analysis. The data showed that there was a slight increase in the expression level of GR in alantolactone-treated cell lysates further confirming that GSH depletion is not linked with its oxidation to GSSG (Figure 7B).

Next we asked if GSH depletion is resulted from GSH extrusion from cells. Therefore, we measured the level of GSH in cells treated with alantolactone for 6 h in the presence of 2 mM methionine, a specific GSH carrier inhibitor. As shown in Figure 7A, the inhibitor of GSH carrier (Methionine) did not prevent GSH depletion, indicating that GSH depletion is not associated with GSH extrusion. To further support this finding, we measured GSH in culture medium. The level of GSH in culture medium was not detectable. The data demonstrate clearly that GSH depletion is not due to GSH extrusion.

Next we treated the cells with 3 mM NAC, a precursor molecule for GSH synthesis for 30 min, followed by treatment with 40 μM alantolactone for 6 h, and intracellular GSH was determined in control and treated cells. As shown in Figure 6A, pretreatment with NAC, completely inhibited the depletion of intracellular GSH. To further exclude the possibility of GSH depletion by inhibition of GSH synthesis, we measured the mRNA expression of γ-glutamylcystein synthetase (γ-GCS) which is a rate limiting enzyme in the synthesis of GSH. As shown in Figure 7C, no change in the mRNA expression of γ-GCS was observed. The data demonstrate that GSH depletion is not associated with inhibition of GSH synthesis. Therefore, the depletion of intracellular GSH by alantolactone is most probably the result of rapid binding of exomethylene moiety of alantolactone with intracellular GSH.

Finally, we incubated 1mM alantolactone with 0, 5, 15 and 30 mM GSH in DMEM medium without FBS at 37°C for 30 min. Following incubation, the samples were analyzed by HPLC (Waters) using XTerra MS C18 (5μm, 4.6×150 mm) column. The mobile phase was composed of acetonitrile (A) and water (B). The gradient program was as follows: 0-30 min, A = 65%, B = 35%. The elution profile was analyzed at 227 nm by UV detector. The data showed that concentration of alantolactone decreased in the presence of increasing concentration of GSH (Figure 7D). The data demonstrate clearly the direct interaction of alantolactone with GSH. Taken together, the data demonstrate

that alantolactone depletes intracellular GSH in HepG2 cells via direct conjugation with GSH.

Alantolactone inhibits STAT3 activation and its downstream target gene Bcl-2

Recent studies show that under oxidative stress, STAT3 is glutathionylated with concomitant inhibition of its phosphorylation. In other words, GSH depletion inhibits STAT3 activation [16]. Because alantolactone reduced the intracellular GSH in HepG2 cells, we were interested if alantolactone could inhibit STAT3 activation. Thus we measured the expression of $pTyr^{705}$ STAT3 in cells. As shown in Figure 8, alantolactone treatment decreased the expression of $pTyr^{705}$ STAT3 in a time-dependent manner. After tyrosine phosphorylation (Tyr^{705}) of STAT3, it translocates into the nucleus where it increase the expression of genes implicated in cell proliferation such as Bcl-2 and cyclin D1. Disruption of STAT3 signalling decreases the expression of anti-apoptotic proteins and induces apoptosis in tumor cells. Therefore, we measured the expression of Bcl-2 family proteins using Western blot analysis. As expected, alantolactone treatment reduced the expression of anti-apoptotic protein Bcl-2 and increased the expression of pro-apoptotic protein Bax in a time-dependent manner (Figure 8).

Alantolactone induces caspase-3 activation in HepG2 cells

Mitochondrial-dependent apoptosis is initiated by recruitment and activation of caspases. Thus we analyzed whether caspase-3 was activated during alantolactone-induced apoptosis of HepG2 cells. As shown in Figure 8, alantolactone stimulated the cleavage of caspase-3 in a time-dependent manner as demonstrated by the appearance of 32 kDa and 17 kDa fragments.

Discussion

Currently available chemotherapy remains ineffective to cure hepatoma mainly because of its high hepatotoxicity. Therefore, alternative therapeutic agents that kill cancer cells without or with low hepatotoxicity are highly desirable. Alantolactone, a sesquiterpene lactone has been reported to possess antibacteria, antifungal, antihelminthic, and anticancer activities. In our previous report, we showed that alantolactone induced

apoptosis in U87 glioblastoma cells via GSH depletion without inducing toxicity in mouse liver and kidneys [15]. However, the molecular mechanism of GSH depletion by alantolactone remained unexplored. The present study was therefore conducted to evaluate whether alantolactone can deplete GSH and induce apoptosis in liver cancer cells. To study the anticancer activity of alantolactone towards liver cancer cells, we used HepG2 cell line as a model cell line. We found that alantolactone inhibited the growth and induced apoptosis in HepG2 cells in a dose- and time-dependent manner as evident by Annexin V positive staining and caspase-3 activation. This alantolactone-induced cell death was completely inhibited when cells were pretreated with NAC, a GSH precursor molecule. The data suggest that GSH depletion might be involved in apoptotic cell death induced by alantolactone. We therefore measured the level of intracellular GSH in control and treated cells. In accordance with our previous study [15], alantolactone reduced the level of GSH in HepG2 cells in a time-dependent manner.

It is well established that intracellular redox status or oxidative stress plays an important role in cancer cells apoptosis [21]. GSH is one of the most abundant intracellular antioxidants involved in the protection of cells against oxidative damage [15, 22]. Depletion of intracellular GSH is an early hallmark in the onset of apoptosis [21, 23]. The intracellular GSH depletion might be resulted either from increased intracellular oxidation of GSH or stimulated GSH extrusion through specific carrier or inhibition of GSH synthesis or direct conjugation of GSH with drug [21]. In the present study, alantolactone-mediated GSH depletion is unlikely to be due to oxidation of GSH into GSSG since expression of GR is not inhibited and the level of GSSG remained unchanged in cells before and after treatment. Furthermore, GSH and GSSG were not detectable in culture medium and methionine (inhibitor of GSH carrier) did not prevent GSH depletion which excludes the possibility of GSH extrusion as the main mechanism for GSH depletion. Pretreatment of cells with NAC completely inhibited GSH depletion and mRNA expression of γ-GCS remained unchanged in control and alantolactone-treated cells. These sets of data demonstrate clearly that alantolactone-induced GSH depletion in HepG2 cells through a process that does not involve inhibition of GSH synthesis. Therefore, the depletion of intracellular GSH by alantolactone is most probably the result of direct conjugation of alantolactone with GSH. Alantolactone like other

sesquiterpene lactones such as parthenolide and helanalin, contains α-methylene-γ-lactone moiety which can interact with sulfhydral group of GSH by means of Michael type conjugation [21] . In order to ascertain the GSH depletion by direct conjugation with alantolactone, we incubated the alantolactone with GSH as described in Materials and Methods and samples were analyzed by HPLC. The data indicated that the amount of alantolactone decreased with the increasing concentration of GSH demonstrating their direct conjugation.

An increasing body of literature evidence underlines the critical role of redox reactions in the regulation of various cell functions. Reactive oxygen species are produced inside the cells during normal physiological processes of the cells which are being neutralized by the antioxidant system of the cells [24, 25]. A precise balance between ROS production and antioxidant system's ability to scavenge ROS is critical for normal cellular functions. GSH is one of the most important intracellular antioxidant which plays major role in protection of cells against oxidative damage. Depletion of GSH results in oxidative stress which is a known inducer of the transcription of specific genes involved in apoptosis [26]. In order to ascertain the mechanism by which alantolactone-induced GSH depletion induces apoptosis in HepG2 cells, we measured the expression of $pTyr^{705}$ STAT3 and its downstream target protein Bcl-2. We found that alantolactone treatment reduced the expression of phosphorylated STAT3 and Bcl-2 in a time-dependent manner. The inhibition of STAT3 by alantolactone is further supported by another recent study that parthenolide a sesquiterpene lactone that depletes GSH in HepG2 cells by direct conjugation with GSH also inhibits STAT3 expression [27] . Both alantolactone and parthenolide share a common α-methylene-γ-lactone moiety which directly conjugates with selfhydral group of GSH. However, the molecular mechanism by which GSH depletion inhibits STAT3 expression remained unexplored in the present study. Further study is needed to bridge the information link between GSH depletion and STAT3 inhibition.

GSH depletion and ROS generation (oxidative stress) are known to act as second messengers to activate diverse redox-sensitive signalling cascades including mitochondrial intrinsic apoptotic cascade through interaction with Bcl-2 family proteins [20, 28]. Bcl-2 family proteins include a wide variety of anti-apoptotic proteins such as

Bcl-2 and pro-apoptotic proteins such as Bax, which are key players of mitochondrial outer membrane permeabilization and apoptosis regulation [20, 29]. GSH depletion and oxidative stress have been reported to activate and translocate pro-apoptotic protein Bax to outer mitochondrial membrane (OMM) where its forms oligomers, which are thought to be important in the formation of permeability transition pores (PTP) and cytochrome c release [20, 22, 30]. Apart from Bax activation, ROS have also been shown to inhibit anti-apoptotic protein Bcl-2 [20, 30]. In the present study, the expression of Bax increased while the expression of Bcl-2 decreased in alantolactone-treated cells in a time-dependent manner. The data demonstrated that alantolactone induced apoptosis in HepG2 cells through mitochondrial pathway. This mitochondrial apoptotic pathway was further confirmed by measuring the mitochondrial membrane potential (MMP) using flow cytometry. A significant reduction in MMP has been observed in alantolactone-treated cells, suggesting the opening of mitochondrial permeability transition pore. Therefore, we concluded that alantolactone can promote the opening of mitochondrial permeability transition pore by increasing the Bax/Bcl-2 ratio.

Conclusion

In conclusion our data provide evidence for the first time that alantolactone depletes GSH in HepG2 cells by direct conjugation with GSH. The GSH depletion resulted in inhibition of phosphorylated STAT3 expression and oxidative stress. Consequently, inhibition of STAT3 activation and oxidative stress induced apoptosis in HepG2 cells by modulating mitochondrial Bcl-2 family proteins. Our results suggest that alantolactone may be a promising chemotherapeutic drug candidate for the treatment of liver cancer. Further investigation is needed to validate the contribution of alantolactone to tumor therapy *in vivo*.

Acknowledgment

This work is supported by Ministry of Education of Pakistan and Chinese Scholarship Council of China.

Conflict of Interests

The authors have declared that no conflict of interest exists.

References

[1] R. J. Thoppil and A. Bishayee, "Terpenoids as potential chemopreventive and therapeutic agents in liver cancer," *World J Hepatol,* vol. 3, pp. 228-49, 2011 .

[2] X. W. Tan, H. Xia, J. H. Xu, and J. G. Cao, "Induction of apoptosis in human liver carcinoma HepG2 cell line by 5-allyl-7-gen-difluoromethylenechrysin," *World J Gastroenterol,* vol. 15, pp. 2234-9, 2009.

[3] L. Andreana, G. Isgro, L. Marelli, N. Davies, D. Yu, S. Navalkissoor, and A. K. Burroughs, "Treatment of hepatocellular carcinoma (HCC) by intra-arterial infusion of radio-emitter compounds: trans-arterial radio-embolisation of HCC," *Cancer Treat Rev,* vol. 38, pp. 641-9, 2012.

[4] A. Alisi and C. Balsano, "Enhancing the efficacy of hepatocellular carcinoma chemotherapeutics with natural anticancer agents," *Nutr Rev,* vol. 65, pp. 550-3, 2007.

[5] T. Beppu, K. Sugimoto, K. Shiraki, M. Tameda, S. Kusagawa, K. Nojiri, J. Tanaka, N. Yamamoto, Y. Takei, H. Takaki, J. Uraki, A. Nakatsuka, K. Yamakado, and K. Takeda, "Clinical significance of tumor markers in detection of recurrent hepatocellular carcinoma after radiofrequency ablation," *Int J Mol Med,* vol. 26, pp. 425-33, 2010.

[6] P. J. Johnson, "Non-surgical treatment of hepatocellular carcinoma," *HPB (Oxford),* vol. 7, pp. 50-5, 2005.

[7] B. Liu, G. Wang, J. Yang, X. Pan, Z. Yang, and L. Zang, "Berberine inhibits human hepatoma cell invasion without cytotoxicity in healthy hepatocytes," *PLoS One,* vol. 6, p. e21416, 2011

[8] T. W. Chung, Y. C. Lee, and C. H. Kim, "Hepatitis B viral HBx induces matrix metalloproteinase-9 gene expression through activation of ERK and PI-3K/AKT pathways: involvement of invasive potential," *FASEB J,* vol. 18, pp. 1123-5, 2004.

[9] W. Y. Wu, J. Li, Z. S. Wu, C. L. Zhang, and X. L. Meng, "STAT3 activation in monocytes accelerates liver cancer progression," *BMC Cancer,* vol. 11, p. 506, 2011.

[10] L. Lin, S. Deangelis, E. Foust, J. Fuchs, C. Li, P. K. Li, E. B. Schwartz, G. B. Lesinski, D. Benson, J. Lu, D. Hoyt, and J. Lin, "A novel small molecule inhibits STAT3 phosphorylation and DNA binding activity and exhibits potent growth suppressive activity in human cancer cells," *Mol Cancer,* vol. 9, p. 217, 2010.

[11] A. Ghantous, H. Gali-Muhtasib, H. Vuorela, N. A. Saliba, and N. Darwiche, "What made sesquiterpene lactones reach cancer clinical trials?," *Drug Discov Today*, vol. 15, pp. 668-78, 2010.

[12] S. Zhang, Y. K. Won, C. N. Ong, and H. M. Shen, "Anti-cancer potential of sesquiterpene lactones: bioactivity and molecular mechanisms," *Curr Med Chem Anticancer Agents,* vol. 5, pp. 239-49, 2005.

[13] H. C. Pal, I. Sehar, S. Bhushan, B. D. Gupta, and A. K. Saxena, "Activation of caspases and poly (ADP-ribose) polymerase cleavage to induce apoptosis in leukemia HL-60 cells by Inula racemosa," *Toxicol In Vitro,* vol. 24, pp. 1599-609, 2010.

[14] A. Trendafilova, C. Chanev, and M. Todorova, "Ultrasound-assisted extraction of alantolactone and isoalantolactone from Inula helenium roots," *Pharmacogn Mag,* vol. 6, pp. 234-7, 2010.

[15] M. Khan, F. Yi, A. Rasul, T. Li, N. Wang, H. Gao, R. Gao, and T. Ma, "Alantolactone induces apoptosis in glioblastoma cells via GSH depletion, ROS generation, and mitochondrial dysfunction," *IUBMB Life,*2012.

[16] E. Butturini, E. Cavalieri, A. C. de Prati, E. Darra, A. Rigo, K. Shoji, N. Murayama, H. Yamazaki, Y. Watanabe, H. Suzuki, and S. Mariotto, "Two naturally occurring terpenes, dehydrocostuslactone and costunolide, decrease intracellular GSH content and inhibit STAT3 activation," *PLoS One,* vol. 6, p. e20174, 2011.

[17] M. Khan, B. Zheng, F. Yi, A. Rasul, Z. Gu, T. Li, H. Gao, J. I. Qazi, H. Yang, and T. Ma, "Pseudolaric Acid B Induces Caspase-Dependent and Caspase-Independent Apoptosis in U87 Glioblastoma Cells," *Evid Based Complement Alternat Med,* vol. 2012, p. 957568, 2012.

[18] M. Khan, B. Yu, A. Rasul, A. Al Shawi, F. Yi, H. Yang, and T. Ma, "Jaceosidin Induces Apoptosis in U87 Glioblastoma Cells through G2/M Phase Arrest," *Evid Based Complement Alternat Med*, vol. 2012, p. 703034, 2012.

[19] X. Ling, Y. Zhou, S. W. Li, B. Yan, and L. Wen, "Modulation of mitochondrial permeability transition pore affects multidrug resistance in human hepatocellular carcinoma cells," *Int J Biol Sci*, vol. 6, pp. 773-83, 2010.

[20] Y. B. Ji, Z. Y. Qu, and X. Zou, "Juglone-induced apoptosis in human gastric cancer SGC-7901 cells via the mitochondrial pathway," *Exp Toxicol Pathol*, vol. 63, pp. 69-78, 2011.

[21] S. Zhang, C. N. Ong, and H. M. Shen, "Critical roles of intracellular thiols and calcium in parthenolide-induced apoptosis in human colorectal cancer cells," *Cancer Lett*, vol. 208, pp. 143-53, 2004.

[22] P. Guha, A. Dey, R. Sen, M. Chatterjee, S. Chattopadhyay, and S. K. Bandyopadhyay, "Intracellular GSH depletion triggered mitochondrial Bax translocation to accomplish resveratrol-induced apoptosis in the U937 cell line," *J Pharmacol Exp Ther*, vol. 336, pp. 206-14, 2011.

[23] M. L. Circu and T. Y. Aw, "Glutathione and apoptosis," *Free Radic Res*, vol. 42, pp. 689-706, 2008.

[24] S. Deng, Y. Yang, Y. Han, X. Li, X. Wang, Z. Zhang, and Y. Wang, "UCP2 inhibits ROS-mediated apoptosis in A549 under hypoxic conditions," *PLoS One*, vol. 7, p. e30714, 2012.

[25] V. Sharma, C. Joseph, S. Ghosh, A. Agarwal, M. K. Mishra, and E. Sen, "Kaempferol induces apoptosis in glioblastoma cells through oxidative stress," *Mol Cancer Ther*, vol. 6, pp. 2544-53, 2007.

[26] C. Kretz-Remy and A. P. Arrigo, "Gene expression and thiol redox state," *Methods Enzymol*, vol. 348, pp. 200-15, 2002.

[27] D. Carlisi, A. D'Anneo, L. Angileri, M. Lauricella, S. Emanuele, A. Santulli, R. Vento, and G. Tesoriere, "Parthenolide sensitizes hepatocellular carcinoma cells to TRAIL by inducing the expression of death receptors through inhibition of STAT3 activation," *J Cell Physiol*, vol. 226, pp. 1632-41, 2010.

[28] Y. Tsujimoto and S. Shimizu, "Role of the mitochondrial membrane permeability transition in cell death," *Apoptosis,* vol. 12, pp. 835-40, 2007.

[29] M. L. Circu and T. Y. Aw, "Reactive oxygen species, cellular redox systems, and apoptosis," *Free Radic Biol Med,* vol. 48, pp. 749-62, 2010.

[30] E. Mayola, C. Gallerne, D. D. Esposti, C. Martel, S. Pervaiz, L. Larue, B. Debuire, A. Lemoine, C. Brenner, and C. Lemaire, "Withaferin A induces apoptosis in human melanoma cells through generation of reactive oxygen species and down-regulation of Bcl-2," *Apoptosis,* vol. 16, pp. 1014-27, 2011.

Figure Legends

Figure 1

Growth inhibition of HepG2 cells after treatment with alantolactone. Cells were cultured for 24 h before drug treatment in 96 well plates. Cells were treated with alantolactone (0,10, 20, 30, 40, 50 and 60 µM) for 12 h and cell viability was measured by MTT assay. Data are expressed as Mean ± SD (n=3). Columns not sharing the same superscript letter differ significantly ($P<0.05$)

Figure 2

Changes in HepG2 cell morphology during alantolactone induced cell death. HepG2 cells were treated with 40 µM alantolactone in the presence or absence of 3 mM NAC for various time intervals and morphological changes were observed by phase contrast microscopy. (A) control, (B,C & D) cells were treated with 40 µM alantolactone for 3, 6 and 12 h, (E) cells were treated with 40 µM alantolactone in the presence of 3 mM NAC for 12 h, (F) cells were treated with NAC alone for 12 h respectively. (G) Cells were treated with 40 µM alantolactone as described above and live and dead cells were quantified using fluorescent probe calcein AM and PI as described in Materials and Methods section. Data are expressed as Mean ± SD (n=3). Columns not sharing the same superscript letter differ significantly ($P<0.05$)

Figure 3

Flow cytometry analysis of Apoptosis in HepG2 cells treated with 40 µM alantolactone for various time points. (A) Control, (B,C & D) Cells were treated with 40 µM alantolactone for 3, 6, and 12 h respectively, (E) Cells were treated with 40 µM alantolactone in the presence of 3 mM NAC for 12 h. (F) Data are expressed as Mean ± SD (n=3). Columns not sharing the same superscript letter differ significantly ($P<0.05$)

Figure 4

Flow cytometry analysis of ROS generation in control and alantolactone-treated HepG2 cells. (A) Control, (B, C & D) Cells were treated with 40 μM alantolactone for 3, 6, and 12 h respectively. After treatment, cells were incubated with DCFH-DA for 30 min at 37°C, washed with PBS and analyzed for DCF fluorescence by flow cytometry. (E) Data are expressed as Mean ± SD (n=3). Columns not sharing the same superscript letter differ significantly ($P<0.05$)

Figure 5

Flow cytometry analysis of MMP in control and alantolactone-treated HepG2 cells. (A) Control, (B, C & D) Cells were treated with 40 μM alantolactone for 3, 6, and 12 h respectively. After treatment, cells were incubated with Rhodamine 123 for 30 min in dark, washed with PBS and analyzed for MMP by flow cytometry. (E) Data are expressed as Mean ± SD (n=3). Columns not sharing the same superscript letter differ significantly ($P<0.05$)

Figure 6

Measurement of intracellular GSH and GSSG in control and alantolactone-treated HepG2 cells for various time points. Cells were cultured in 6 well plates and treated with or without 40 μM alantolactone for 3, 6 and 12 h. Intracellular GSH and GSSG were measured according to the instructions of kit. Data are expressed as Mean ± SD (n=3). Columns not sharing the same superscript letter differ significantly (P<0.0

Figure 7

Measurement of GSH in the presence of various inhibitors and expression of genes involved in GSH metabolism. (A) HepG2 cells were treated with 40 μM alantolactone for 6 h in the presence or absence of various inhibitors and concentration of GSH was measured according to kit instructions. Data are expressed as Mean ± SD (n=3). Columns not sharing the same superscript letter differ significantly ($P<0.05$). (B) Cells were treated

with 40 μM alantolactone for indicated time points and cell lysates were subjected to Western blot analysis for the expression of glutathione reductase (GR). (C) Cell were treated with 40 μM alantolactone for indicated time points and mRNA expression of γ-glutamyl cysteine synthetase (γ-GCS) was determined by RT-PCR. (D) Alantolactone (1 μM) was incubated with indicated concentrations of GSH in medium for 30 min and amount of alantolactone was assessed using HPLC. The data shows that GSH decreased the amount of alantolactone in a dose-dependent manner. Data are expressed as Mean ± SD (n=3). Columns not sharing the same superscript letter differ significantly ($P<0.05$).

Figure 8

Effect of alantolactone on the expressions of apoptosis regulators. HepG2 cells were treated with or without 40 μM alantolactone for 6 and 12 h. The expression of Phospho-STAT3, Bax, Bcl-2 and caspase-3 was measured by Western blot analysis.

Figure 1

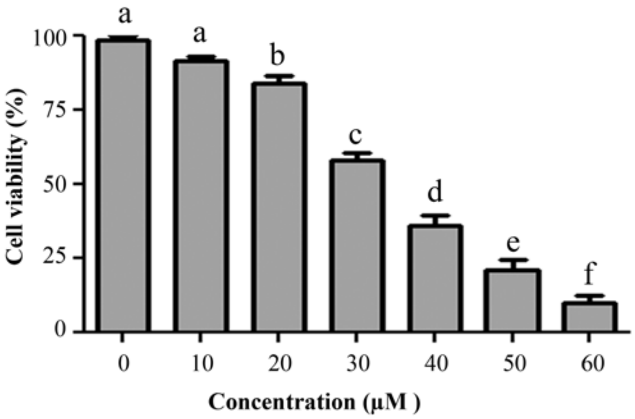

a
a
b
c
d
e
f
100
75
50
25
0
Cell viability (%)
0
10
20
30
40
50
60
Concentration (µM)

Figure 2

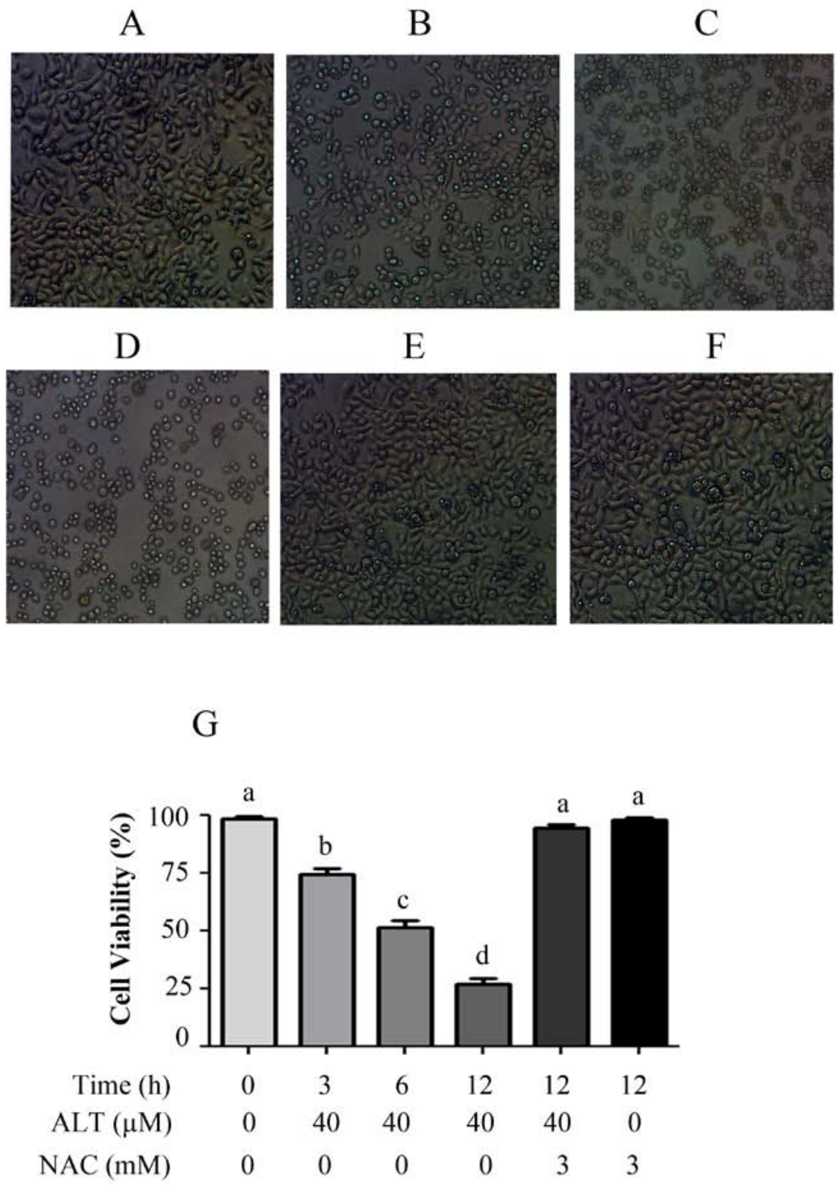
A
B
C
D
E
F
G
Cell Viability (%)
100
75
50
25
0
a
b
c
d
a
a
Time (h) 0 3 6 12 12 12
ALT (μM) 0 40 40 40 40 0
NAC (mM) 0 0 0 0 3 3

Figure 3

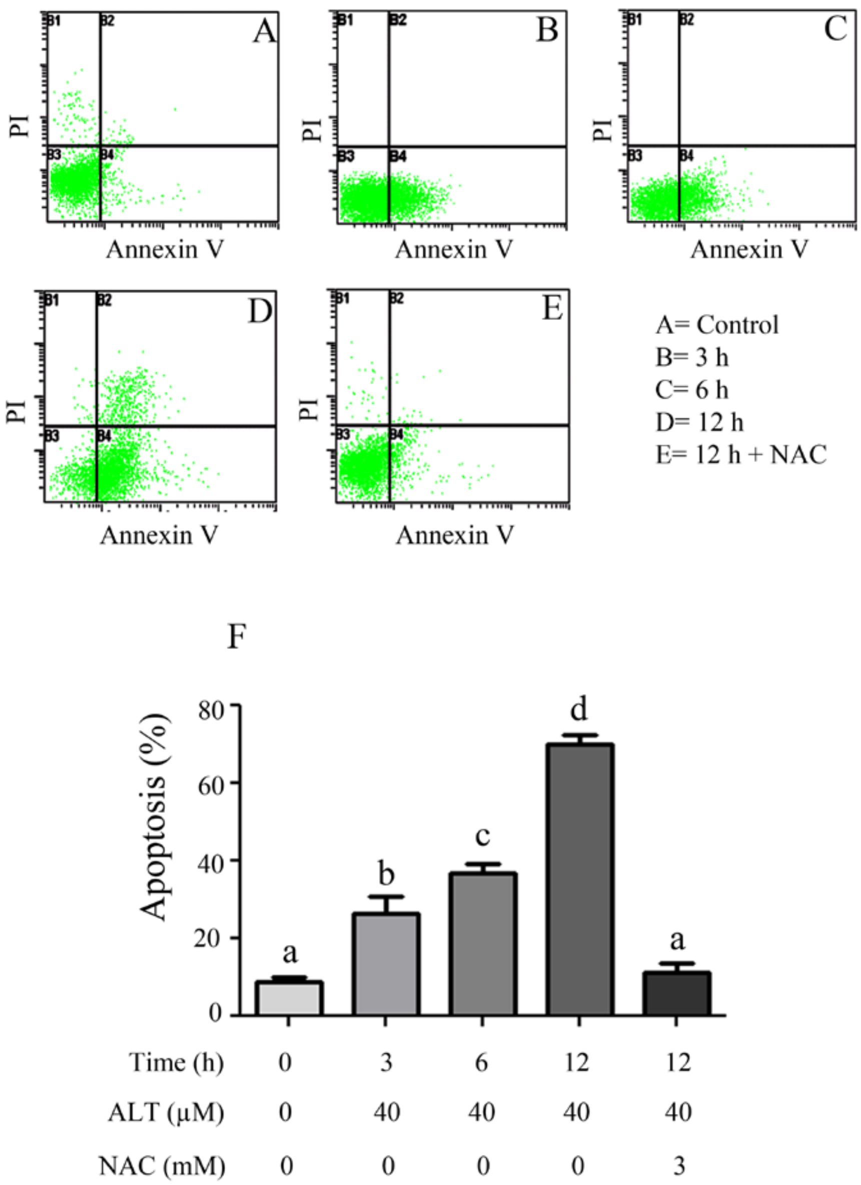

B1
B2
B3
B4
A
B
C
D
E
PI
Annexin V
A= Control
B= 3 h
C= 6 h
D= 12 h
E= 12 h + NAC
F
Apoptosis (%)
80
60
40
20
0
a
b
c
d
a
Time (h) 0 3 6 12 12
ALT (μM) 0 40 40 40 40
NAC (mM) 0 0 0 0 3

Figure 4

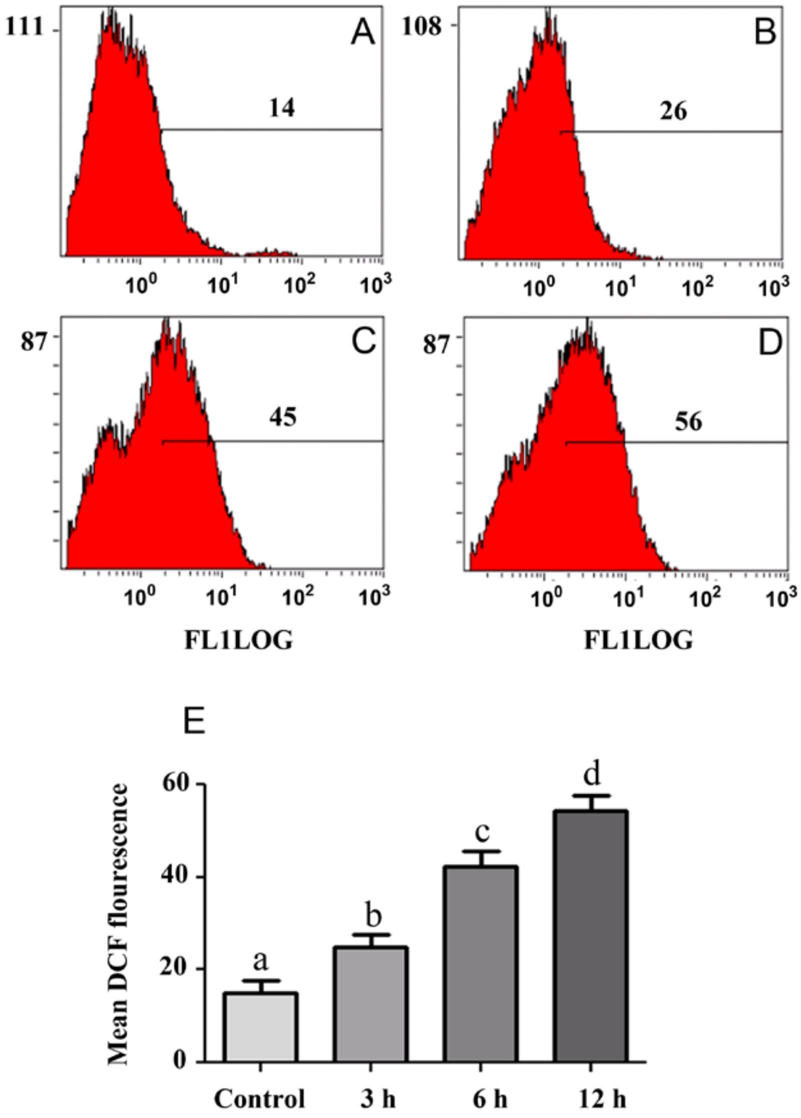

111
A
14
108
B
26
87
C
45
87
D
56
10^0 10^1 10^2 10^3
FL1LOG
FL1LOG
E
Mean DCF flourescence
60
40
20
0
a
b
c
d
Control
3 h
6 h
12 h

Figure 5

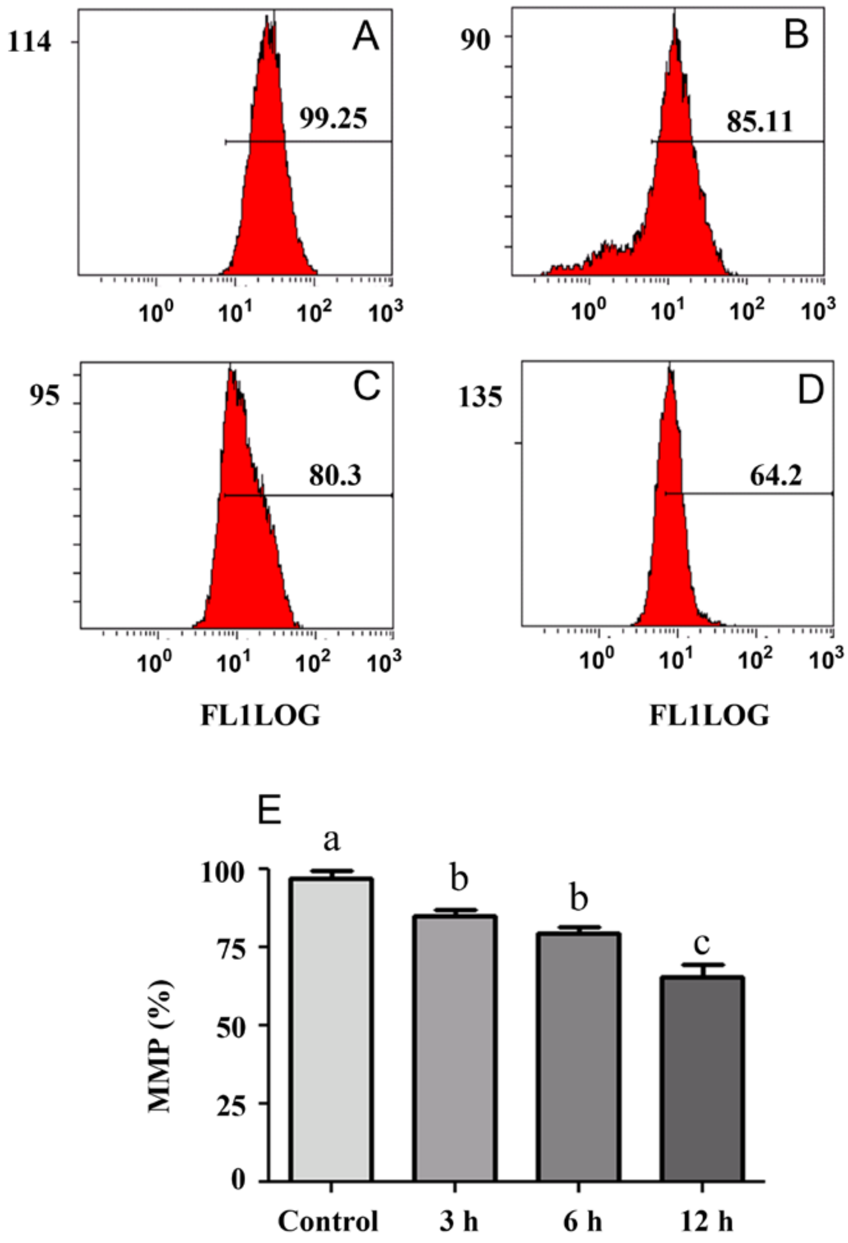

A
114
99.25
B
90
85.11
C
95
80.3
D
135
64.2
10^0 10^1 10^2 10^3
FL1LOG
FL1LOG
E
MMP (%)
0
25
50
75
100
a
b
b
c
Control
3 h
6 h
12 h

Figure 6

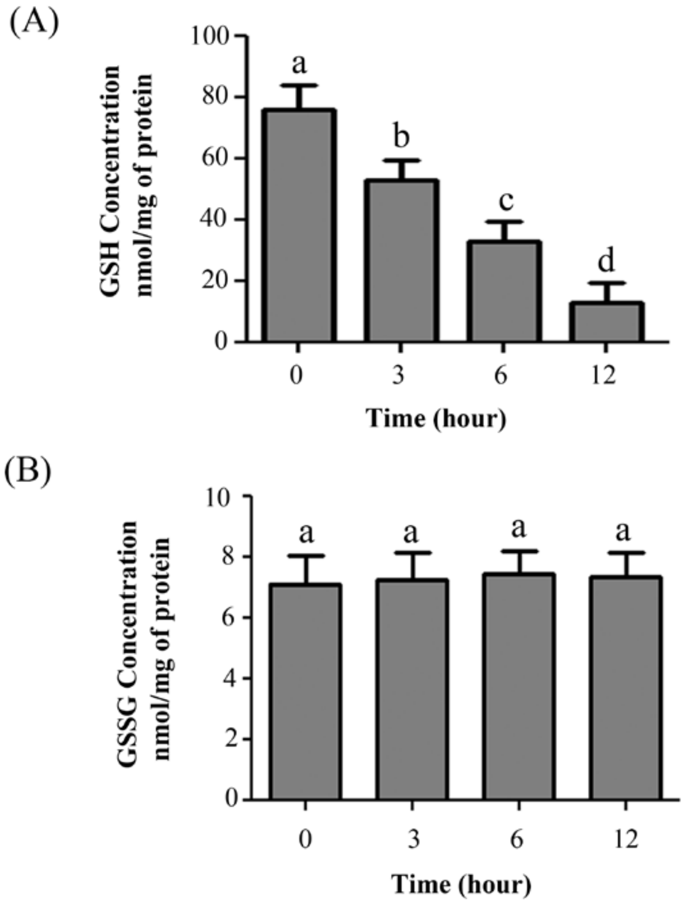

(A)
GSH Concentration nmol/mg of protein
100
80
60
40
20
0
a
b
c
d
0
3
6
12
Time (hour)
(B)
GSSG Concentration nmol/mg of protein
10
8
6
4
2
0
a
a
a
a
0
3
6
12
Time (hour)

Figure 7

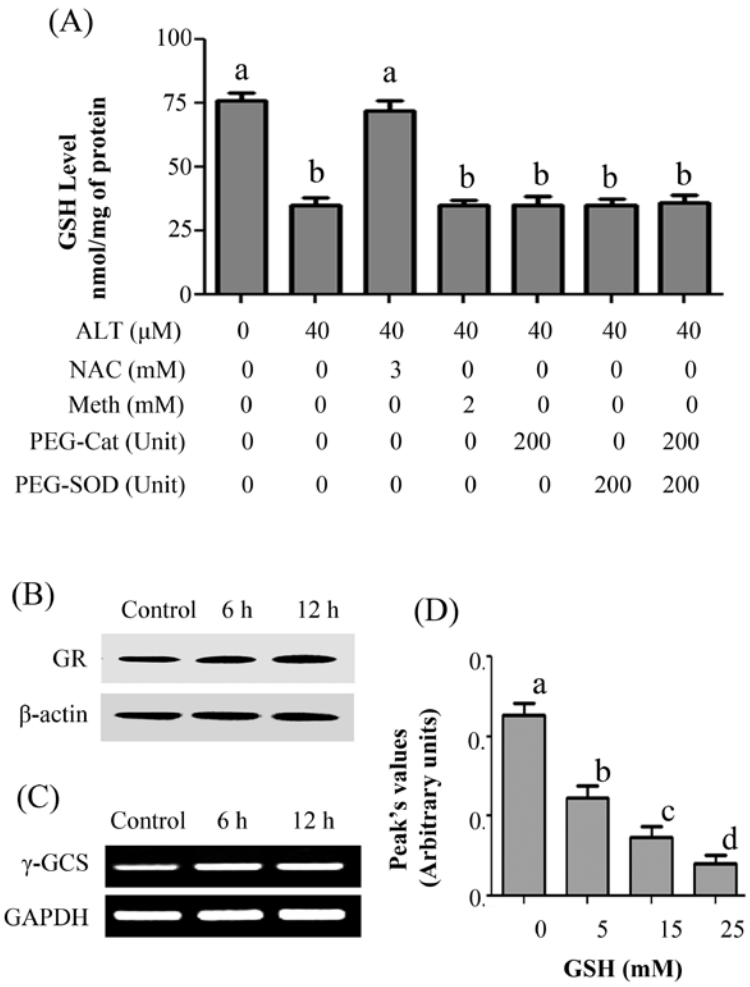
(A)
GSH Level
nmol/mg of protein
100
75
50
25
0
a
b
a
b
b
b
b
ALT (μM) 0 40 40 40 40 40 40
NAC (mM) 0 0 3 0 0 0 0
Meth (mM) 0 0 0 2 0 0 0
PEG-Cat (Unit) 0 0 0 0 200 0 200
PEG-SOD (Unit) 0 0 0 0 0 200 200
(B)
Control 6 h 12 h
GR
β-actin
(C)
Control 6 h 12 h
γ-GCS
GAPDH
(D)
Peak's values
(Arbitrary units)
a
b
c
d
0 5 15 25
GSH (mM)

Figure 8

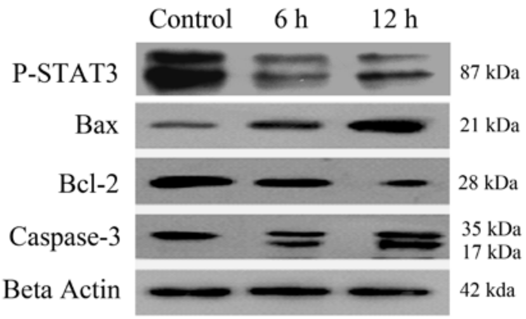
Control
6 h
12 h
P-STAT3
87 kDa
Bax
21 kDa
Bcl-2
28 kDa
Caspase-3
35 kDa
17 kDa
Beta Actin
42 kda

Supplementary Data

Figure 1

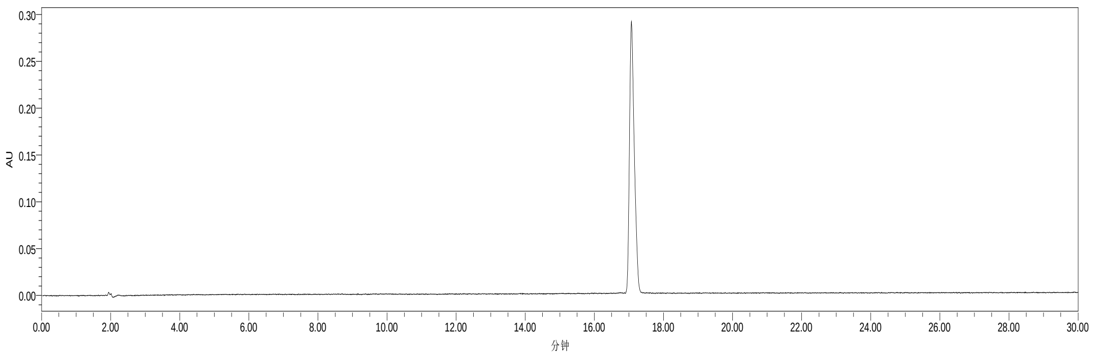

Purity of Alantolactone (99.12%) was determined by HPLC. HPLC were performed on a Waters XTerra C18 (4.6mm×150mm, 5μm). The mobile phase consisted of (A) acetonitrile containing 0.2% acetic acid and (B) 0.2% acetic acid in water with gradient elution: 0–15 min, 10–70% B; 15–30 min, 70–80% B; The temperature of autosampler was maintained at 25 ◦C and detection wavelength was 254nm.
